# Does standing or sitting position of the anesthesiologist in the operating theatre influence sevoflurane exposure during craniotomies?

**DOI:** 10.1186/s12871-016-0284-0

**Published:** 2016-12-01

**Authors:** Péter Sárkány, Béla Tankó, Éva Simon, Judit Gál, Béla Fülesdi, Csilla Molnár

**Affiliations:** Faculty of Medicine, Department of Anesthesiology and Intensive Care, University of Debrecen, H-4032 Debrecen, Nagyerdei krt. 98., Hungary

**Keywords:** Occupational exposure, Sevoflurane

## Abstract

**Background:**

Exposure of the OR staff to inhalational anesthetics has been proven by numerous investigators, but its potential adverse effect under the present technical circumstances is a debated issue. The aim of the present work was to test whether using a laminar flow air conditioning system exposure of the team to anesthetic gases is different if the anesthetist works in the sitting as compared to the standing position.

**Methods:**

Sample collectors were placed at the side of the patient and were fixed at two different heights: at 100 cm (modelling sitting position) and 175 cm (modelling standing position), whereas the third collector was placed at the independent corner of the OR. Collected amount of sevoflurane was determined by an independent chemist using gas chromatography.

**Results:**

At the height of the sitting position the captured amount of sevoflurane was somewhat higher (median and IQR: 0.55; 0.29–1.73 ppm) than that at the height of standing (0.37; 0.15–0.79 ppm), but this difference did not reach the level of statistical significance. A significantly lower sevoflurane concentration was measured at the indifferent corner of the OR (0.14; 0.058–0.36 ppm, *p* < 0.001).

**Conclusions:**

Open isolation along with the air flow due to the laminar system does not result in higher anesthetic exposure for the sitting anesthetist positioned to the side of the patient. Evaporated amount of sevoflurane is below the accepted threshold limits in both positions.

**Electronic supplementary material:**

The online version of this article (doi:10.1186/s12871-016-0284-0) contains supplementary material, which is available to authorized users.

## Background

Exposure of the OR (operating theatre) staff to inhalational anesthetics has been proven by numerous investigators using different techniques, but its potential adverse effect under the present technical circumstances (low- and minimal flow anesthesia systems, modern air conditioning and scavenging systems in the int he OR) is a debated issue [1]. Although threshold-limit concentrations for sevoflurane, the most frequently used anesthetic, have been defined for some countries, these limits are not uniform and are arbitrarily defined, merely based on animal studies [1].

There are some observations among OR-personnel suggesting central nervous complications –such as headaches, dizziness, memory problems, fatigue, or attention problems at the end of the working day [2, 3], while others made the observation that under the recommended level of exposure CNS symptoms are not more frequent [4]. Elevated liver enzymes were recently documented in health workers exposed to anesthetic gases [5] and the impact of chronic exposure on reproduction (decreased feritlity, spontaneous abortions and congenital abnormalities) cannot be excluded [6], either. In view of these observations preventive measures are taken to reduce occupational exposure of the OR staff to the minimum.

It was recently documented that, during craniotomies, occupational exposure for the anesthesia team is higher than that for the operating neurosurgeon and the main source of sevoflurane evaporation is the patient’s mouth [7]. In a previous study it was also proven that positioning of anesthesia at the feet of the patient (rather than at the side) may decrease anesthetic exposure [8]. However, in some institutions anesthetists insist on positioning anesthesia team at the side because, in their judgment, the patient is better accessible this way. However, as is known from previous studies, laminar flow air conditioning systems – although they more potently decrease sevoflurane concentrations in the OR in general - direct air flow toward the anesthetist and consequently, exposure may be higher [9].

As we run a laminar flow air conditioning system in our OR –according to the newest standards-, the aim of the present work is to test whether exposure of the team to anesthetic gases is different if the anesthetist works in the sitting as compared to the standing position.

## Methods

We included 27 patients (12 females and 15 males) undergoing craniotomy for the removal of intracerebral tumors. All patients signed an informed consent approved by the local medical ethics committee (University of Debrecen, Health and Medical Science Center), registration number of institutional ethics approval was: DEOEC RKET/IKET 2483-2006, responsible person: Dr. József Szentmiklósi).

**Fig. 1:**
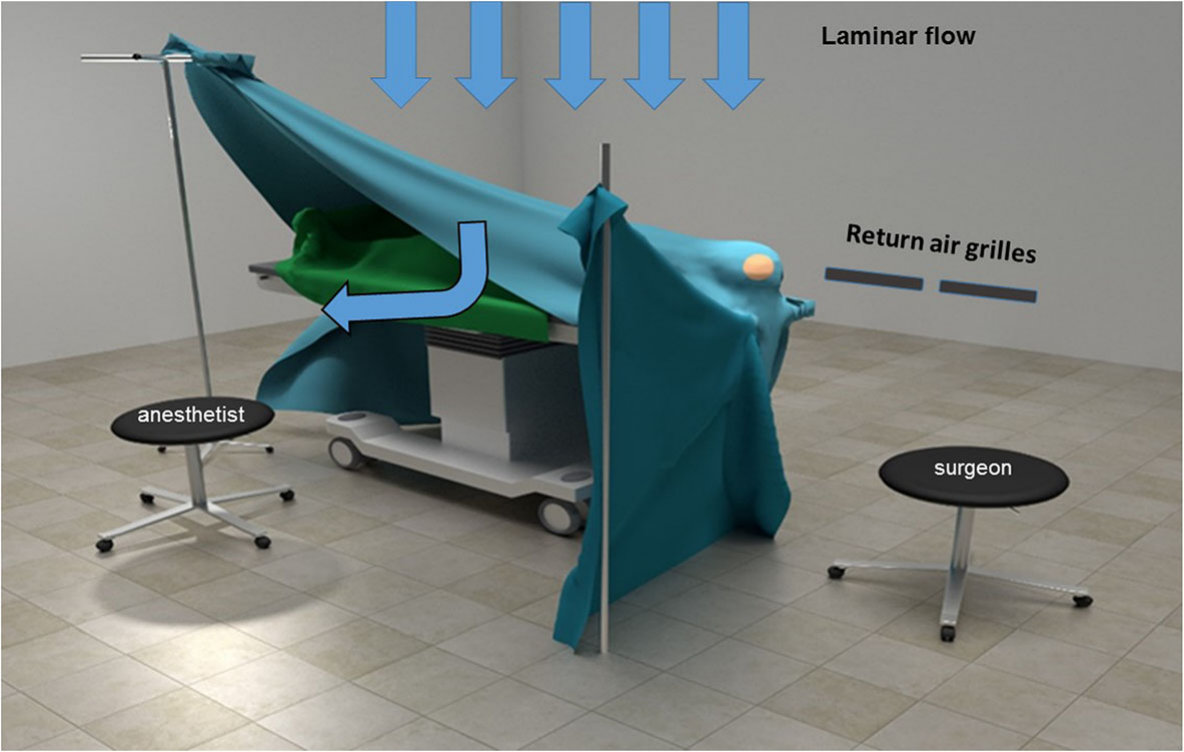
The position of the patient, neurosurgeon and the anesthesia team during craniotomies with an illustration of the laminar flow air system

For anesthetic induction propofol (1-2,5 mg/kg BW) and for maintenance a combination of sevoflurane, fentanyl androcuronium was used. Sevoflurane was administered via a Dräger Zeus anesthesia work station (Dräger Medical AG & Co. KG, Lübeck, Germany) using a low-flow anesthetic technique. Tracheal tubes were inflated using pressure gauge to 25–30 mmHg pressure Routine perioperative monitoring was used including arterial blood pressure, heart rate, ECG, O2 saturation, end-tidal CO2 concentration and end-tidal sevoflurane concentration. The anesthesia team were positioned at the side of the patients as shown in Fig. 1. Craniotomy and dural opening were always started after equilibrium of sevoflurane anesthesia was reached (usually at 1 V% sevoflurane). A laminar flow air conditioning system was used allowing changing and refilling the air within the OR at a rate of 50 m^3^/min.

To collect the evaporated amount of sevoflurane a setup was used consisting of a portable air sampling pump (224-51 TX Air Sampling Pump SKC, Dorset, England), an attached tube system and an absorber ampule coupled to the tube system; the details of the sampling were described elsewhere in detail [7]. The suction pump ensured a continuous flow through the absorber ampule where sevoflurane was collected for later analysis using gas chromatography. Sample collection was started at skin incision and terminated at dural closure. Sevoflurane concentration could be determinded as a time weighted average. Time weighted average sevoflurane concentration was calculated accoring to the following equation=

$$ \frac{{\mathrm{V}}_0*\ \mathrm{m}*\ {10}^6}{\mathrm{M}*\mathrm{Q}*\mathrm{t}} $$

where

V_0_ = the volume of 1 mol sevoflurane at room temperature (24 l)

m = quantity of sevoflurane in the absorber

M = quantity of 1 mol sevoflurane (200,055 g)

Q = minute volume of the suctioning pump (300 ml/min)

t = duration of sample collection (minutes).

To answer the present study’s question, three detectors were placed at the different sites of the OR as follows: an infusion stand was placed at the side of the patient and sample collectors were fixed at two different heights: at 100 cm (modelling sitting position) and 175 cm (modelling standing position), whereas the third collector was placed at the independent corner of the OR. This type of positioning of the sample collectors were chosen in order to exclude the impact of movement of the anesthetist during surgery (changing positions, going away from the anesthesia working place) and therewith being able to assess the influence of the position in the circumstances of the laminar flow air conditioning system. Additional sample collectors were placed on the floor near to the return air grilles and at the mouth of the patient, but their results were not part of the present statistical analysis carried out to answer our question, they provided additional information.

### Statistical analysis

After normality tests failed, medians and interquartile ranges are reported for all values. Pairwise multiple comparisons were performed by the appropriate Tukey-test. A *p* < 0.05.value was accepted as statistically significant difference.

## Results

**Fig. 2:**
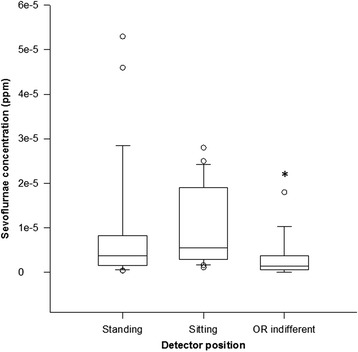
Sevoflurane concentrations (medians and IQR) at the height of sitting and standing position with the team located on the side of the patient as well as at the indifferent corner of the OR. * indicates statistically significant difference compared to values measured at sitting and standing position

Demographic characteristics of the patients are summarized in Table 1. Results on the collected amount of sevoflurane are summarized in Fig. 2. At the height of the sitting position the captured amount of sevoflurane was somewhat higher (median and IQR: 0.55; 0.29–1.73 ppm) than that at the height of standing (0.37; 0.15–0.79 ppm), but this difference did not reach the level of statistical significance. A significantly lower sevoflurane concentration was measured at the indifferent corner of the OR (0.14; 0.058–0.36 ppm, *p* < 0.001) Values of each measurements are presented as an additional file.

**Table 1:** Patients and operation characteristics

	Parameter
Age (years)	50.1 ± 16.8
Body mass index	25.6 ± 6.2
Gender (F/M)	12/15
Duration of surgery, minutes	108.7 ± 45.8
ET CO_2_	30.3 ± 1.9
ET sevoflurane (vol%)	1.39 ± 0,38

Sevoflurane concentrations measured at the patient’s mouth were gradually higher than at any other points of the OR (1.2; 0.43–7.1 ppm). Absorbers placed on the floor near the return air grilles collected 0.26; 0.16–0.94 ppm sevoflurane.

## Discussion

Occupational exposure of the OR team to anesthetic gases cannot be avoided and long-term consequences on the personnel’s health are not known in all details [1, 10]. Therefore attention should be paid to measures that decrease the hazard of exposure . Among other things, type of surgical intervention, technique of the inhalational anesthesia, airway device used during surgery, positioning of the anesthesia team as well as type and capacity of the the air-conditioning and scavanging system are factors that can modify anesthetic exposure [1, 9].

In the present study we checked the hypothesis that during craniotomy operations, with the team positioned at the side of the patient the standing position results in lower exposure of the team than the sitting position. The concept of this hypothesis arose from the observation of Herzog-Niescery et al. [9], who stated that using a laminar flow air-conditioning system may lead to higher exposure of the anesthetist than using a turbulent system. We therefore hypothesited that, as shown in Fig. 1, when a laminar system is used, the gas flow is driven toward the anesthetist located at the side of the patient. As we proved previously during our measurements, the main source of sevoflurane evaporation is the patient’s mouth [7] and the proximity of the anesthetist located at the side contributes to higher exposure due to the open isolation drape driving the evaporated gas toward the anesthetist [8]. Therefore, the open isolation along with the air flow due to the laminar system may result in higher anesthetic exposure for the sitting anesthetist (See Fig. 1). Although this concept was not proven by our investigations due to a lack of statistically significant differences in sevoflurane concentrations at the height of sitting and standing, it has to be noted that both median and especially upper IQR were much higher at the height of the sitting position. Of note also that the measured values were below the accepted threshold limits of sevoflurane exposure (2 ppm).

It is important to point out that our measurements were performed after an intravenous induction and maintenance occurred with intratracheal inhalational anesthesia, therefore our results are not generalizable. For sake of clarity, we started collecting the evaporated amount of sevoflurane after the induction and endotracheal intubation (after skin incision), when the steady-state was reached. On purpose we intended to exclude the effect of higher pollution occuring during induction (intravenous induction was used, followed by inhalational maintanance), because we wanted to assess the effect of standing or seating position. Previously many authors have reported on higher amounts of evaporated anesthetic gases when laryngeal masks were used [9, 11] or during inhalational inductions [9, 12]. As the amount of sevoflurane that escapes through the mouth of the patients in intratracheal anesthesia is much lower than that observed during administration of a laryngeal mask airway, it is conceivable that the exposure differences between the sitting and standing positions may be clinically important in certain scenarios.

The most important question of all evaporation study is how much difference in exposure would there need to be for this difference to be important? There is lack on sufficient data to answer this question. Although threshold limit concentration for anesthetic gases are defined and taken into account in some countries, but they are arbitrarily defined. In fact, there are reports on potential long-term side effects in exposed workers [3, 5, 6], but systematic, long-term follow-up studies under the present technical circumstances (low flow-minimal flow technique, air flow systems in the ORs) are lacking. Further studies are needed to answer this question.

## Conclusion

In conclusion: there are many open questions and debated issues related to the occupational exposure of inhaled anesthetics. Although the time-weighted average is usually below the arbitrarily defined threshold limits, long-term effects on occupationally exposed workers are not fully known. Therefore, technical measures and organisatory aspects have to be taken in order to minimize the potential hazards of anesthesia teams. These may include positioning of the anesthesia team to the feet of the patients during intracranial surgeries, as suggested previously (8) or placing a fan to the breating zone of the anesthesia team in case of laminar flow air conditioning system. Recent results suggest that beside the technical environment, local behavioural factors in the operating theatre may influence anesthetic exposure [13].

## Additional file

Additional file 1: Values of each measurements. (XLSX 9 kb)

## Abbreviations

OR: Operating room
ppm: Par per million
